# “*I don’t mind damaging my own body”* A qualitative study of the factors that motivate smokers to quit

**DOI:** 10.1186/1471-2458-15-4

**Published:** 2015-01-21

**Authors:** Jane Bethea, Barnaby Murtagh, Susan E Wallace

**Affiliations:** Department of Health Sciences, The University of Leicester, Adrian Building, University Road, LE1 7RH Leicester, UK

**Keywords:** Smoking, Cessation, Knowledge, Smoking related disease, Chronic obstructive pulmonary disease, Risk, Motivation

## Abstract

**Background:**

Although smoking prevalence in England has declined, one in five adults smoke. Smokers are at increased risk of a number of diseases, including COPD which affects an estimated 1.5 million people in England alone. This study aimed to explore issues relating to smoking behaviour and intention to quit that might be used to inform the development of cessation interventions. Issues explored included knowledge of smoking related disease, with a particular emphasis on Chronic Obstructive Pulmonary Disease (COPD). Understanding around risk of disease, including genetic risk was explored, as were features of appropriate and accessible cessation materials and support.

**Methods:**

Semi-structured interviews and focus groups were conducted with a total of 30 individuals of which 17 were smoking cessation clients and 13 were professionals working within health care settings relevant to supporting smokers to quit. A largely purposive approach was taken to sampling, and data were analysed using the constant comparative method.

**Results:**

Knowledge of the smoking related disease COPD was limited. Smokers’ concerns around risk of disease were influenced by their social context and were more focussed on how their smoking might impact on the health of their family and friends, rather than how it might impact on them as individuals. Participants felt the provision of genetic risk information may have a limited impact on motivation to quit. Genetic risk was considered to be a difficult concept to understand, particularly as increased risk does not mean an individual will definitely develop disease. In terms of cessation approaches, the use of visual media was consistently supported, as was the use of materials that linked directly with life experiences. Images of children inhaling second hand smoke for example, had a particular impact.

**Conclusions:**

Public health messages around the risks of smoking and approaches to quitting should continue to have an emphasis on the dangers that an individual’s smoking has on the lives of the people around them. More work also needs to be done to raise awareness around both the risk of COPD in smokers and the impact this disease has on quality of life and life expectancy.

## Background

Smoking represents a significant public health burden to individuals, communities and healthcare systems across the world. The burden of disease from smoking, including mortality, is most pronounced in low and middle-income countries but is also a significant problem in developed countries [1]. Smoking is also a key cause of health inequality. In men aged 35–69 years, approximately one-half of socioeconomic differences in mortality are attributable to smoking [2].

The World Health Organization estimate that six million people die from smoking related causes each year [3]. It is estimated that in England two out of every ten deaths in adults aged 35 and over are as a result of smoking [4]. Smoking is associated with increased risk of a number of diseases such as lung, oral pharyngeal and breast cancer. It is also closely associated with risk of the respiratory disease Chronic Obstructive Pulmonary Disease (COPD), where an estimated 73% of deaths are related to smoking [5].

COPD has a significant impact on quality of life [6]. Symptoms of the disease include breathlessness, cough and wheezing and people living with condition report that coping with symptoms and associated exacerbations becomes a ‘way of life’ [7]. It is estimated that in England alone there are 1.5 million people living with COPD [8], and a significant number of these will be current or ex-smokers. However despite this, there is evidence to suggest that awareness of the disease among smokers is poor in comparison to diseases like lung cancer [9]. Although smoking has been described as the most important cause of COPD [10], genetic variation also plays a part in an individual’s risk of developing the disease [11]. As such, smokers with a greater genetic susceptibility will be at an even higher risk of developing COPD.

Smoking behaviour is influenced by social context. It has been reported that in the UK, young girls and children of white British origin are at a greater risk of smoking [12]. Children whose parents smoke are also at increased risk of smoking in adulthood [13]. Smoking prevalence is also associated with socio-economic status, and in England approximately one quarter of people in routine and manual households smoke, compared to 13% of those living in households classified as being managerial/professional [14].

Those living in deprived communities may face particular challenges in terms of smoking cessation, as it has been shown that they are more likely to be in pro-smoking environments where smoking is normalised [15, 16]. Although cessation attempts do not vary across socio-economic groups, people in more deprived communities are less successful in their quit attempts [17]. It is possible that smoking in these environments may facilitate social inclusion within the community and so may isolate members from positive health promoting norms [16].

Smoking cessation is an important focus of many health care systems and in the UK smokers wishing to quit can access free of charge support from services provided by the National Health Service (NHS). These widely promoted services can be accessed through the smoker’s GP or through self-referral. Smokers are offered support for a period of twelve weeks and this includes one to one or group support sessions with a stop smoking adviser. At these sessions clients receive advice on coping with cravings and avoiding relapse, and are also offered a breath test that measures the level of carbon monoxide present in their body. Clients can also choose to access a range of nicotine replacement therapies including lozenges or patches, as well as prescribed medications (bupropion or varenicline). Smokers not wishing to access this service can receive support for cessation from their GP.

Cessation interventions, including those in the UK, are commonly underpinned by the transtheoretical model of behaviour change also known as the ‘stages of change’ model [18]. The model suggests that chronic behaviours can be categorised into one of five stages: pre-contemplation, contemplation, preparation, action and maintenance [19]. In terms of smoking cessation this means that once an individual has made the decision to stop smoking, they move from the contemplation and preparation stages into action, which may include accessing a smoking cessation service. By providing nicotine replacement therapy, the service aims to assist the individual through the action stage and into long term maintenance.

Large scale media campaigns also form part of government funded tobacco control interventions in the UK. Such campaigns have been shown to have a positive effect on smoking in adults, both in terms of reducing smoking prevalence and overall cigarette consumption [20, 21]. A study of media campaigns in England reported that messages relayed in these campaigns tend to focus on the negative consequences of smoking [22]. However, a study of the emotive content of campaigns found that compared to negatively emotive messages, positively emotive messages had a bigger impact on the number of smokers calling a cessation telephone ‘quit line’ [23].

However, despite having access to a range of smoking cessation interventions, people continue to smoke. Reasons for this might include that smokers tend to downplay the risks smoking poses to their health [24, 25] and at the same time overly emphasise perceived benefits such as stress relief [25]. Smokers may also generally underestimate their own risk compared to that of the average smoker [26, 27].

The public health challenge is to develop and deliver smoking related interventions that are effective and take into account socially contextualised motivators and barriers to successful cessation. The aim of this study was to explore what both smokers and professionals working with smokers thought were the key factors that influenced the decision to stop smoking. This included understanding of smoking related disease, with a particular emphasis on COPD. Understanding of risk and how genetic risk of a smoking disease such as COPD would impact on smoking behaviour were also explored, as were views on what works in terms of smoking cessation initiatives.

## Methods

The study design and conduct were informed by the RATS guidelines [28]. A qualitative approach, using focus groups and semi-structured interviews was used to allow participants to express their feelings and experiences regarding strategies to stop smoking. Prior to data collection, appropriate ethical and organisational approval was obtained for research done in the UK NHS system. A literature review was conducted and examples of smoking cessation materials available through the NHS, as online resources or as smartphone applications, were collected and reviewed in order that they could be used as examples when discussing aids with participants. To gain a better understanding of both smoking and smoking cessation, a member of the study team (BM) also shadowed a designated core worker from a NHS smoking cessation service. This involved observing a number of smoking cessation appointments and talking to workers about the content and structure of a typical cessation programme.

### Recruitment

In order to answer our research question, a mixed sampling approach was adopted. In terms of client participants, smoking cessation clinic venues were selected purposively according to their client profile and local demographics. Three venues were identified by the local NHS smoking cessation service. The first was in an area of high deprivation with a predominantly White British population and an older age profile than that of the city as a whole. The second was in an area with a similar age profile but with low levels of deprivation and an ethnically diverse population. The final site was in an area of ethnic diversity, but with high levels of deprivation and a younger age profile than the city as a whole.

Once the clinic venues were selected, clients were eligible for inclusion if they were current or ex-smokers and aged 18 years or over. Initially a smoking cessation advisor in the three clinic venues introduced the study to eligible clients and then those interested were asked to provide their contact details to the study team. It was the intention that potential participants would be selected for inclusion according their characteristics, including age, gender and ethnicity. However, only five cessation clients responded using this approach. As a result, an ethics amendment was sought to allow the study researcher (BM) to recruit directly from clinic venues. Clients were then approached largely on an opportunistic basis although the researcher actively attempted to ensure that both male and female clients were recruited. Overall 17 clients agreed to be interviewed and five agreed to participate in a group discussion. Of these 12 were female and five were male.

In addition to client participants, 13 professional participants were also recruited. Potential professional participants were identified purposively according to their role and included those working with NHS smoking cessation services or in relevant area of health service delivery. General Practitioners and Practice Nurses were recruited to take part in a focus group discussion or an interview through a local primary care training event. One additional GP and a Practice Nurse with a specific interest in COPD were identified through a local COPD focussed conference. A hospital based Consultant working in the area of respiratory care and two senior pharmacists with specific interests in respiratory illness were also identified through this event. Members of a local COPD strategy group also participated in a group discussion.

### Data collection

Data were collected through semi-structured interviews and focus groups. Four focus groups were conducted in total, two with professionals and two with smoking cessation clients. In addition, 16 one to one interviews were conducted with four professional participants and 12 client participants. Although no cash payment was provided to participants, client participants were offered a £20 high street gift voucher as a thank you for taking part in the study. Data were collected between November 2012 and June 2013.

All interviews were conducted by BM who does not have a clinical, public health or smoking cessation background. Focus groups and group discussions were conducted primarily by BM and moderated by JB and/or a member of academic staff employed by the University of Leicester. All interviews and group discussions were audio-recorded with the agreement of the participant(s) and all participants gave written informed consent prior to data collection.

A topic guide was used to facilitate data generation and participants were asked to share:

- Views on smoking and smoking cessation, including what influences decision to quit
- Understanding of smoking related disease, with a particular emphasis on Chronic Obstructive Pulmonary Disease (COPD)
- Views and experiences (both positive and negative) of different smoking cessation interventions, including views on the suitability and usefulness of different types of media and the use of novel approaches.

### Data analysis

All interviews were audio-recorded and transcribed verbatim. Analysis was iterative and informed by the constant comparative method [29]. Preliminary open codes were generated from each focus groups and then emerging ideas were tested, developed and refined throughout the subsequent focus groups and one to one interviews. Primary analysis was done by BM and shared and discussed with JB to ensure appropriate reporting and interpretation. Any disagreement or requirement for clarification was resolved by JB and BM returning to the transcribed data and exploring how the issue/interpretation under discussion was evidenced by the data collected.

## Results

Analysis of the data identified several key themes common across smoking cessation clients and professionals. Relationships with children and the wider family and also finances acted as key motivators in deciding to change smoking behaviour. Risk to themselves as a smoker had little impact on decision to change behaviour, but the impact of their behaviour on the risk of causing illness in others held much more weight. Clients also had limited knowledge or understanding of COPD, and what knowledge they did have tended to be through the lived experience of friends or family members who had been diagnosed with the disease.

### The impact smoking has on others as a motivating factor to quit

Client participants consistently reported that relationships with either their children, or with other family members, were primary drivers in their decision to stop smoking or reduce the amount they smoked. This was consistent with the views of professional participants. Those who were ex-smokers felt this had influenced their own smoking behaviour and could also be used as a lever to promote cessation in others:

*Agnes: …there was the advert with children involved in smoke and that did have an impact on me. I didn’t smoke around children… no, that did have an impact.*

*Harriet: I’d concur with that. It’s what hit home with me to stop smoking.*

*Agnes: Yeah … that really hit home powerfully. But yeah it was the connection with my own children.*

*(Agnes and Harriet, professionals who are ex-smokers working in the field of smoking cessation)*

When asked about the harm that smoking caused, the consensus was that smokers understand and accepted that they are harming their own bodies. They were, however, far more concerned that their actions could be harming others, particularly children:

*“More than what you would on your health reasons. Cos everybody’s not really, apart from the health fanatics, nobody’s actually bothered about their health are we?” (Adam, Client participant).*

and

*“I think it’s as well I don’t mind damaging my own body, I do that quite successfully actually, but yeah I think it’s the fact that (it has) an impact on your children I think always to me has a great, you know, as a mother it has a greater impact I think” (Agnes, Professional participant and ex-smoker).*

This was directly counterpointed by smokers without children who stated that messages around the impact of smoking on children had no meaning to them:

*“See I don’t have children so it had no impact on me what so ever. I saw it, but it didn’t really mean anything to me, because I don’t have children so again I think it’s because people relate it to their own lives don’t they?” (Allan, professional participant and ex-smoker).*

As well as being fearful of exposing children and family members to harm through second hand smoke, participants were also aware that smoking impacted negatively upon their ability to participate in family life:

*“Especially now you’ve got a grandson you see it more like missing out on like his walking or talking or you might miss that first word because you’ve nipped out for a cigarette”. (Belinda, client participant)*

### Smoking as a financial burden

The financial burden associated with smoking was for some an additional factor to health concerns that triggered a desire to quit, whilst for others health had very little bearing and the decision was a purely financial one:

*“Money. Both money and health reasons. Cos I was smoking quite a lot. I was on about fifty to sixty a day” (Felix, client participant)*

and:

“*…some people pack up for health reasons but whatever reasons to pack up for, I’m trying to save money I’m smoking for the bedroom tax, so… if you pack up smoking you can get yourself a nice car or pay for your bedroom tax”. (Adam, client participant)*

Professionals working with smokers also raised financial costs, both in the short and long term, as reasons why their patients or clients had made the decision to stop smoking. One professional participant (a General Practitioner) reported an incident where he had successfully and perhaps almost unintentionally acted as a direct trigger for a patient to stop smoking. This has been achieved by linking an important health message to that patient’s life and goals for the future, including the patient’s wishes to have a good retirement:

*“…financial issues help. I know that I told, someone came to me for a medical 25 years ago for a pension, life insurance and I said ‘oh’ I said ‘you must love your wife very much.’ He said ‘why do you say that?’ I said ‘well you’re smoking 20 a day you’re not going to be around to pick it up’, So I didn’t see him for about 20 years, forget why he came in..So he said ‘not since the day I came in for my medical, I stopped that day’.*

Interviewer: Successful point of contact.

“*It was relevant. I’m saving for my retirement and I’m not going to be around to pick it up. What the hells the bloody point?” (Borris, professional participant)*

### The impact of health messages and advice

There was an agreement between healthcare professionals and smoking cessation service clients that smokers were unlikely to take notice or would be sceptical of the merits of health care advice around the risks of smoking unless that advice directly affected their life or the lives of people they knew and cared for.

Resistance to health advice in the form of media campaigns again was associated with how the message impacted upon the clients lived experience and on their ability to link the information presented to their own life. This was associated with the ability to link with other people’s real–life experiences and successes in giving up smoking, although these also had to be credible and ‘real’. The use of actors, for example, to relay even true stories was thought to have limited impact as it was still ‘not real’:

*“Being cynical I could say well that’s an actor, even though I know it’s real. But like I think no that could be an actor. That’s not a real person, so you’re talking rubbish sort of thing”. (Deirdre, client participant)*

For some the resistance to health information was associated to feeling bombarded with often very negative images related to smoking and other lifestyle related issues such as obesity. These had lost meaning to these clients who actively ignored them:

*“You just think oh well another one. That’s what it’s getting like. Every time you turn the telly on there’s something about health things, either smoking or losing weight….” (Adam, client participant).*

### Understanding of smoking related disease

In terms of understanding of disease, discussion of smoking related disease tended to be orientated around cancer and not around other important diseases like COPD. The client participants generally had a poor understanding of this specific disease. Again knowledge was associated with experience, and those who had some basic knowledge about COPD predominantly already had either emphysema or chronic bronchitis, or knew someone with these diseases:

*“I’ve seen people with COPD, so I know how poorly they can be. It does make you worry, it really does”. (Grace, client participant)*

In addition to broad health messages around smoking cessation, both clients and professionals were asked their views on whether the provision of information to smokers that included personalised genetic risk of a smoking disease, such as COPD, would impact on smoking behaviour. Again there was resistance to this from clients in that it was thought to be difficult to understand, particularly in understanding that increased risk of a disease does not mean a person will definitely go on to develop that disease, or that any change in lifestyle would guarantee avoiding it. This was further compounded by having friends or family who despite being non-smokers had still gone on to develop diseases often associated with smoking:

*Francine: There’s always going to be things like that with genetics. Some people will think yeah genetic make-up could be a high risk of having cancer, this, that and the other. Change your lifestyle you might not get it, but who’s to say you could get it or you couldn’t?*

*Edmond: … they always say cancer is a lot to do with smoking. I’ve known people that don’t smoke, never have done in their life, never been in a smoke environment, but died of lung cancer. Arguments over everything. Especially where genetics is concerned. I do admit it’s good on some things like one illness me daughter’s got that is a genetic thing, but on other things when do you say that it is genetic or not? And I don’t think knowing would help”. (Edmond and Francine, client participants)*

### The delivery of cessation messages

Participants were also asked about their views and experiences of smoking cessation interventions, both in terms of the form of media used and in the key messages that were relayed. Consistently, visual media was seen as having both the biggest impact and being the most accessible approach to relaying information:

*“I think, I know a lot of our patients, they’re not readers, and so leaflets isn’t so valuable but a visual impact they will carry with them which might be helpful”. (Gina. Professional participant).*

The use of visual media, in particular new media technologies such as interactive mobile applications, online videos and games were seen as vehicles which provide ways to engage smokers around future health risks. The participants felt that such approaches may be particularly helpful in preventing initiation of smoking in young people, but also felt that they may have less impact on older smokers:

*“Apps are OK for younger generations and internets are OK for the younger generation. Older generation they have no idea how to go in. I tell patients to go and look at the NHS Choices site, they don’t. They always complain they couldn’t get through to it. So these things will take maybe another generation for them to use it well”.* (*Larry. Professional participant)*

And

“*There’s this thing, isn’t there, whereby they take your photo as you are now and then they show you a picture of you in, I don’t know, 15/20 years’ time if you carry on smoking and that’s, I think especially for young girls especially, that would be quite an eye opener to say, “Look, you’re going to have these lines and this sagginess as well as smelling awful.” I think that’s a really good… that part of it was really good but obviously that was since I’ve stopped smoking but visually it’s quite powerful. This is what will happen you know and I think using new technologies is probably… how you do it I don’t know but if you’re using new technologies like that it’s quite effective, especially with younger people.” (Katie. Client participant)*

As discussed earlier, the potential physical harm to children and loved ones was a powerful influence on some smokers’ decision to quit. In terms of cessation materials and the impact of these, the emotional harm that smoking has on children was also seen as important lever:

*“And the one that they had not long ago was children or a child would come up on the TV and he would say, “Dad, I want you to live” and it was so emotional. You’d watch it and at the end he’d say, “Please quit smoking” and you’d think oh God, I should quit [laughs]”.*

*(Gilly. Client participant).*

## Discussion

### Principle findings

The findings of this study suggest that tackling the health burden of smoking is not merely a matter of solving a physical addiction and that smoking cessation approaches and related information needs to take into account an individual’s lived experience and their social context. They also suggest that public health messages that rely on individual health outcomes may have less impact than those that illustrate the effect of an individual’s behaviour on others, particularly children and family members. Furthermore, in terms of knowledge around the health implications of smoking, there appears to be a general lack of understanding around the full range of smoking related diseases. Cancer seemed to be well understood but COPD, which is highly associated with smoking, was not. Also this study suggests that risk as a general concept may be difficult for some smokers to understand or conceptualise. This may be compounded by having friends or family who despite being non-smokers, have developed smoking related disease.

### How this study fits

This study found that risk of smoking related disease tended to be considered in terms of how smoking impacts on risk of ill-health in others, particularly children and family members. Impact on children’s health in particular has been reported as being a key motivator in either the decision to quit smoking or to have a smoke free home [30]. However, a study of smokers in a similar disadvantaged inner city area found that knowledge was variable around the dangers of second hand smoke and that the impact of smoking on the smell and décor of the house were bigger motivators than the health consequences for their children [31]. A systematic review of interventions targeted at parental cessation found that interventions were only effective for parents of children over 4 years of age and not for younger children and babies [32]. Why smokers do not change their behaviour to minimise their risk and also the risk for those around them may be associated with levels of social support. A smoker who is motivated to quit or to limit exposure of children to second hand smoke may still find their efforts negated by a partner who is unwilling to change their behaviour [30].

In this study, personal health outcomes and risk of developing disease tended be discussed in relation to luck. This was rooted in experience of having family or friends who did not smoke but still went on to develop diseases commonly associated with smoking, and vice versa. This could reflect a general underestimation of personal risk in smokers [26, 27, 33] or a degree of misunderstanding around key risk factors for disease [26]. Weinstein and colleagues, in a large scale survey of smokers and non- smokers, reported that smokers tend to underestimate their own risk of lung cancer compared to the ‘average smoker’. They also found that smokers who did not intend to quit were significantly more likely to report that a person’s genetics were primarily responsible for lung cancer development [26].

In addition to seeing their risk of developing smoking related disease as being influenced by luck, the client participants were more likely to talk about health risks associated with smoking in relation to cancer. Knowledge of COPD, which is also highly associated with smoking, was very limited. This lack of knowledge has been reported elsewhere in a qualitative study of patients at risk of COPD, who saw lung cancer as the primary risk to their health [9]. Again in the current study, although overall knowledge was poor, it was better among those that had experience of the disease either personally or through friends and family.

Participants were asked to consider how the provision of information on genetic risk of a condition such as COPD might impact on motivation to stop smoking. This was raised with participants as there are technologies available that can demonstrate how risk of COPD is influenced by genetic variation, and how this risk changes according to smoking status. The ‘Risky Gene Machine’ for example is a web-based application that shows how COPD risk is influenced by both genetic variation and smoking status [34], and it is possible that visual media such as this could be used with smokers as part of a cessation programme.

The evidence around the impact of providing genetic risk information on health related behaviour is though limited [35]. Although the provision of such information does not seem to promote a fatalistic approach to health and related behaviours [36], how it impacts on actual behaviour is unclear. Marteau and colleagues in a systematic review concluded that providing DNA based disease risk estimates may have an impact on intention to change behaviour, but does not lead to actual behaviour change in the short or long term [35]. Why is unclear, although the participants in our study felt that this would be a complex message to relay and understand. Also, this complexity appeared to be linked with fact that the risk information provided may not be absolute – i.e. an increased risk does not mean that an individual would definitely develop disease, and quitting smoking may not mean an individual would definitely not develop disease.

In terms of communicating cessation messages, participants in this study felt that visual media may be particularly effective in delivering key cessation messages. Again, participants tended to refer to media campaigns that highlighted the impact smoking had on others, particularly children. Campaigns that included ‘real’ people also held more weight. Such approaches have been found to have a positive impact. In the United States a large scale smoking cessation campaign called ‘Tips from former smokers’ uses very visual emotive advertisements and real-life stories. The people involved have been affected by smoking, some have never smoked but have illnesses caused or triggered by smoking, whilst others are ex-smokers suffering from diseases such as throat cancer or COPD. In terms of impact, the programme reached a large proportion of smokers (just under 80% recalled seeing at least one of the Tips advertisements) and was associated with an estimated 1.64 million quit attempts, an absolute increase of 3.7% [37].

### Strengths and weaknesses of the study

A particular strength of this study was the diversity of participants in that both the professional and client perspective was sought and also in the overall number of smokers that contributed to the study. In addition, the study recruited largely client participants through clinics orientated around more deprived communities and as such may reflect the views and experiences of participants from communities where smoking behaviours are more normalised and so more entrenched.

The main limitation of this study is that with the exception of a small number of professionals who were also smokers, we accessed current and ex-smokers through a NHS smoking cessation service. This means that the findings presented may not represent the views and experiences of people who quit without support from such a service, or smokers with no agenda to quit. In addition, it proved more difficult than anticipated to recruit smoking cessation clients. As a result, it was not possible to take a truly purposive sampling approach with this group. More women than men participated, and we were unable to recruit participants from a range of ethnic backgrounds.

## Conclusion

Although overall smoking prevalence in England has declined in recent years, it continues to vary according to socio-economic status. This may be because cessation approaches are struggling to successfully engage these smokers at the pre-contemplation stage. Based on the findings of this study, those in lower socio-economic groups might place greater value on the impact that their smoking has on other people rather than themselves. This group might also benefit from a greater understanding of the health impacts of smoking outside of those associated with risk of cancer. More work needs to be done on risk communication strategies, as smokers may struggle to both conceptualise risk and underestimate their own risk of smoking related disease.

Finally taking into account the potential importance of social context and networks, the development and delivery of smoking cessation interventions may also benefit from increased input from smokers and users of smoking cessation services. On a community level, involved individuals become carriers of new knowledge and can have a positive impact within their community by introducing this to their friends and relatives. On a wider level, greater involvement could help ensure that cessation approaches have a focus that reflects key motivating factors that are relevant to the target group.

### Statement of ethics approval

Ethics approval was obtained from the NRES Committee East Midlands – Leicester (reference 12/EM/0240) and organisational approval from Leicestershire Partnership Trust (reference HESRO598). The study was sponsored by The University of Leicester.
